# Chromosomal Location of Traits Associated with Wheat Seedling Water and Phosphorus Use Efficiency under Different Water and Phosphorus Stresses

**DOI:** 10.3390/ijms10094116

**Published:** 2009-09-18

**Authors:** Hong-Xing Cao, Zheng-Bin Zhang, Cheng-Xu Sun, Hong-Bo Shao, Wei-Yi Song, Ping Xu

**Affiliations:** 1 Center for Agricultural Resources Research, Institute of Genetic and Developmental Biology, CAS, Shijiazhuang, 050021, China; E-Mails:hongxing1976@163.com (H.-X.C.);xupingsjz@yahoo.com (P.X.); 2 State Key Laboratory of Soil Erosion and Dryland Farming on the Loess Plateau, Institute of Soil and Water Conservation, Chinese Academy of Sciences, Yangling 712100, China; 3 Coconut Research Institute, Chinese Academy of Tropical Agricultural Sciences, Wenchang, Hainan 571339, China; E-Mail:Suncx@live.com (C.-X.S.); 4 Yantai Institute of Coastal Zone Research for Sustainable Development, Chinese Academy of Sciences, Yantai 264003, China; 5 Institute of Life Sciences, Qingdao University of Science& Technology, Qingdao266042, China; 6 Biology Department, Shangqiu Normal University, Shangqiu 476000, China; E-Mail:songweiyi@163.com (W.-Y.S.)

**Keywords:** chromosomal location, phosphorus use efficiency, seedling stage, wheat, water use efficiency, drought, wheat breeding

## Abstract

The objective of this study was to locate chromosomes for improving water and phosphorus-deficiency tolerance of wheat at the seedling stage. A set of Chinese Spring-Egyptian Red wheat substitution lines and their parent Chinese Spring (recipient) and Egyptian Red (donor) cultivars were measured to determine the chromosomal locations of genes controlling water use efficiency (WUE) and phosphorus use efficiency (PUE) under different water and phosphorus conditions. The results underlined that chromosomes 1A, 7A, 7B, and 3A showed higher leaf water use efficiency (WUE_l_ = Pn/Tr; Pn = photosynthetic rate; Tr = transpiration rate) under W-P (Hoagland solution with 1/2P), -W-P (Hoagland solution with 1/2P and 10% PEG). Chromosomes 7A, 3D, 2B, 3B, and 4B may carry genes for positive effects on individual plant water use efficiency (WUE_p_ = biomass/TWC; TWC = total water consumption) under WP (Hoagland solution), W-P and -W-P treatment. Chromosomes 7A and 7D carry genes for PUE enhancement under WP, -WP (Hoagland solution with 10% PEG) and W-P treatment. Chromosome 7A possibly has genes for controlling WUE and PUE simultaneously, which indicates that WUE and PUE may share the same genetic background. Phenotypic and genetic analysis of the investigated traits showed that photosynthetic rate (Pn) and transpiration rate (Tr), Tr and WUE_l_ showed significant positive and negative correlations under WP, W-P, -WP and -W-P, W-P, -WP treatments, respectively. Dry mass (DM), WUE_P_, PUT (phosphorus uptake) all showed significant positive correlation under WP, W-P and -WP treatment. PUE and phosphorus uptake (PUT = P uptake per plant) showed significant negative correlation under the four treatments. The results might provide useful information for improving WUE and PUE in wheat genetics.

## 1. Introduction

Drought stress is the most important and common environmental issues which limits agricultural production and decreases the efficiency of dry lands [1–3]. Irrigation is commonly applied to alleviate water deficiency, but with the increasing worldwide water resource crisis, no more water can be used for irrigation.

Fertilizer is the second important element for enhancing crop yield. Phosphorus is often considered the most limiting nutrient for plant growth in soils, especially in dry land [4,5]. The main reason may be the lower phosphorus use efficiency (PUE) of crops, although total phosphorus (P) in the soil may be high, it is often present in unavailable forms or in forms that are only available outside of rhizosphere [6,7], meanwhile, large amounts of P fertilizer and poorly managed irrigation systems may lead to P accumulation and pollution of surface and ground waters. To obtain available P from soil, one method is by adding P fertilizer in soil, the second method is by planting high PUE crop varieties [8,9].

In most arid and semi-arid areas, there is more poor land with water and nutrient stress, nutrient absorption cannot be maximized under water stress conditions, and poor nutrients also greatly limit crop water use efficiency (WUE) and yield. Therefore, wheat cultivars with high WUE and PUE are becoming a prerequisite for lowering production costs [10–14]. However, the definition of WUE and PUE differs according to the context in which it is considered, and it is variously defined by agronomists, experts of plant physiology, irrigation engineers and economists. WUE has been defined as the leaf WUE (leaf photosynthetic rate per transpiration rate), whole plant WUE (the ratio of aboveground biomass or dry matter per unit area, and water use by crop); yield WUE (crop grain per unit area, to the transpiration loss from the crop) [15]. P use efficiency (PUE) was classified as shoot (SPUE) or whole plant (WPUE) efficiency, shoot P uptake per plant (SPUT, value of shoot dry weight × total shoot P concentration) and shoot P use efficiency (SPUE, shoot dry weight produced by unit SPUT); whole plant P uptake per plant (WPUT, value of whole plant dry weight × total whole plant P concentration) and whole plant P use efficiency (WPUE, whole plant dry weight produced by unit WPUT) [16–18].

To make progress towards this objective, many studies related to WUE and nutrient use efficiency and their interactions have been done at both the physiological and molecular levels. The variable WUE and nutrient use efficiency of different crops or varieties under different water and nutrient level have been studied [19–22], and these reports showed that water and nutrient uptake are two physiological processes that interact with each other. There has also been much research done on the chromosomal location of these traits in different studies. Morgan used 21 cultivar substitution lines of Chinese Spring/Red Egyptian to locate a single recessive gene for high osmoregulation on chromosome 7A of Chinese Spring [23]. Chromosomes 3A, 3D, 4A and 4D carry genes with increased Pn in *Lophopyrum elongatum (Host) A.L(o)ve* in Chinese Spring background [24]. The genes associated with high leaf water use efficiency (WUE_l_) were located on chromosomes 5A and 5D when the substitute line Chinese Spring-Egyptian Red was the tested material [25]. An effect of 1BL.1RS on drought tolerance was detected. ‘Mv5791-1B.1R’ and the sister line ‘Mv5791-1B.1B’ of rye were investigated, and the results showed that 1RS translocation line could increase HI and WUE under drought conditions [26]. The Pavon 1RS translocation lines had larger root biomass than Pavon 76 bread wheat and could absorb more water–nitrogen solution [27].

The substitution lines derived from the triticale ‘Presto’ and ‘Rhino’ cultivars were tested in hydroponic culture for nitrogen and phosphorus uptake and utilization efficiency. The nitrogen utilization efficiency (NUE) was significantly increased in all substitution lines with the exception of 1D(1R) ones, phosphorus utilizations were generally significantly positive only in the lines 2D(2R) and 6D(6R) [28]. Chromosomes 1D, 2D, 2B, 3A, 3D, 4A, 4B, 6A, 6B, 7D and 7A were associated with drought tolerance of Synthetic 6x in substitution lines of Chinese Spring (CS)-Synthetic 6x [29]. These results indicated that WUE and NUE may share the same genetic background at a molecular level. It is possible to improve drought and nutrient-deficiency tolerance of wheat through genetic approaches [30].

Wheat (*Triticum aestivum* L.), one of the most important crops in the world, is mainly cultivated in arid and semi-arid areas, and always suffers from water and nutrient deficiency [31–33]. Therefore, understanding the genetic control of P-deficiency tolerance and drought resistant is very important to provide strategies for development of higher water and nutrient-efficient cultivars. To our knowledge, there is a little information about the simultaneous chromosomal location concerning WUE and PUE in wheat under different treatments. In this study, the substitution lines derived from Chinese Spring and Egyptian Red were selected to locate genes associated with WUE and PUE on the specific chromosomes under different water and nutrient conditions with the following objectives: (i) to understand the differences of location results of PUE and WUE under different treatments; (ii) to assess the chromosomes simultaneously associated with WUE and PUE; (iii) to determine the relationships between traits; (iiii) to provide information useful for genetically improving WUE and PUE in wheat.

## 2. Results and Discussion

### 2.1. Chromosomal location of the genes associated with Pn (photosynthetic rate) and Tr (transpiration rate)

Pn and Tr values of the considered substitution lines for every treatment are shown in Table 1. Most genotypes showed higher Pn and Tr under WP treatment, a slight decrease under -WP conditions and greatly reduced values under W-P, -W-P treatment.

Under WP (control) treatment, all the A genome and chromosomes 7D, 3D, 6D, 7B, 5D, 6B, 2D, 4D, 1B showed significantly increased Pn compared to the two parents, and the parent CS had the lowest Pn among all the tested materials. Chromosomes 7A, 4A, 1A, 2A, 6A, 3A, 5A, 6D, 4D, 7D, 1D, 5D showed significantly higher Tr, but chromosomes 5B and 3B showed lower Tr than that of the two parents.

Under -WP treatment, chromosomes 7A, 6A, 3A, 5A, 4A, 6D, 7D, 2D, 2A, 3D, 4D had significant increased Pn than that of the two parents, chromosomes 5B, 4B, and 2B had a little lower Pn value compared with the two parental strains. Chromosomes 7A, 1A, 3A, 6A, 5A, 5D, 6D, 2D, 4A, 7D, 2A showed significant increased Tr and chromosomes 5B, 2B, and 4B had lower Tr than that of two parents.

Under W-P conditions, the parent ER showed the highest Pn value, and chromosomes 6A, 4D, 6D, 7D, 7A showed significantly increased Pn compared to CS and chromosome 3B showed a significantly decreased Pn compared to the two parents. Chromosomes 4D, 3D, 2D, 1D had significantly increased Tr compared to the two parents, while chromosomes 7B, 1A, and 6B showed lower Tr than that of the two parents, but only showed significant difference with ER.

Under -W-P treatment, chromosomes 7A, 7D, and 2A showed increased Pn compared with the two parents, and chromosomes 1B, 3D, 4D, 5B, 1D, 3B, 4B, 5D showed significantly decreased Pn compared with the two parents. Chromosomes 2D, 1D, 4A, 6D, 5D had significantly increased Tr and chromosomes 1B, 6B, 4B, 5B, 1A, 3A, 3B, 2B, 7B, 5A, 7A showed significantly decreased Tr compared with the parents. As shown in Table 1, genes with positive effects on Pn and Tr were mainly located on the A and D genome, and genes with negative effects on Pn and Tr on the B genome under different water and P stress conditions.

### 2.2. Chromosomal location of the genes associated with WUE_l_ (Leaf water use efficiency) effects

It can be seen from Table 2 most genotypes showed greater WUE_l_ under -WP conditions. Under WP condition, chromosomes 3B, 6B, 1B, 7B, 3D, and 2B may carry genes with positive effects and chromosome 1A with negative effects on WUE_l_, because they had higher and lower WUE_l_ than that of two parents, respectively. But WUE_l_ of chromosomes 3B, 6B, 1B, 7B, 3D, and 2B showed a significant difference for CS, and WUE_l_ of chromosome 1A showed a significant difference for ER.

Under -WP treatment, chromosomes 4D, 6B, and 2B had higher and chromosomes 1A, 5D, 5B, 2D, 7A, 3A, 4B, 6D, 1B, 5A, 1D, 2A, 6A, and 3D had lower WUE_l_ than that of the two parents, respectively. Only the value of WUE_l_ on chromosomes 1A and 5D showed any significant differences compared to the two parents.

Under W-P condition, chromosomes 4A, 7A, 1A, 6A, 7B, 6B, and 3A had higher and chromosomes 3D, 5D, 2D, 3B, 4D,1D, 6D,1B, 7D, 4B, 5A, and 2B had lower WUE_l_ than that of the two parents.

Only chromosomes 4A, 7A, 1A,3D, 5D, 2D, 3B, 4D,1D, 6D,1B, 7D showed significant differences compared to the two parents.

Under -W-P treatment, the higher WUE_l_ and lower WUE_l_ were observed on chromosomes 2A, 1A, 6B, 7A, 7D, 3A, 7B, 6A, 4B, 2B, 3B, 5A and 1D, 2D, 3D, 4D, 5D, 6D, 4A than that of the two parents, respectively. These substitute lines of chromosomes 2A, 1A, 6B, 7A, 7D, 3A, 7B, 6A, 1D, 2D, 3D, 4D, 5D showed significant differences for both parents.

### 2.3. Chromosomal location of the genes associated with DM (dry mass) TWC (total water consumption)

Most genotypes showed high DM under WP treatments. Higher TWC values were found under WP (control) or W-P treatments (Table 3).

Under WP conditions, chromosomes 5A, 3D, and 4B had higher DM than that of the two parents. Chromosomes 1B, 7B, 1A, 1D, and 6A showed lower DW than that of the two parents, but only showed significant difference with one parent CS or ER. The two parents showed significantly higher TWC than all the other genotypes except 5B.

Under -WP conditions, chromosome 7D showed higher DM, and chromosomes 2D, 1A, and 7B showed lower DM than that of the two parents, but only showed a significant difference with one parent CS or ER, respectively. Higher TWC values were found on chromosomes 6D, 3D, and 4D and lower TWC on chromosomes 7B, 3A, 5A, 1D, 1B, 5D, 1A, 4A, 2D, 6B, 7A, 2A, 2B, 3B, 4B, 7D and 6A compared with the two parents, but only the values of TWC on chromosomes 7B, 3A, 5A, 1D and1B showed any significant differences compared with both parents simultaneously.

Under W-P conditions, chromosomes 1A, 7A, 2B, 3D, 4D, 3B, 7D and 6B were found to have higher DM and lower one was seen on chromosomes 2A, 5D, 4A, 7B, 6A, 1B, and 1D than that of the two parents, and only the DM values on chromosomes 1A and 7A showed any significant differences with the two parents. Chromosomes 5A, 1D, 6D, 4D, 7A, 7D, 2B, 3D, 6A and 1A had positive effect and chromosomes 5B, 5D, 1B and 3A negative effect on TWC, respectively, but only the values of TWC on chromosomes 5A, 1D, 6D, 4D and 7A had any significant difference with the two parents.

Under -W-P treatment, chromosomes 3D, 2B, 1D, 2A, 3B, 7A, 2D, 5A, 6A, 1A and 4B had higher DM than the two parents. Chromosomes 5D and 5B showed lower DM than the two parents; chromosomes 3A, 4D, 6B, 1B, 2D, 6D, 3B, 1D, 7A, 5D, 2B, 5B, 6A, 7B, 3D, and 7D had higher and chromosomes 4B, 2A, 4A, and 5A had lower TWC values than the two parents, respectively. None showed significant differences except the values for chromosomes 3A, 4D, and 6B.

### 2.4. Chromosomal location of the genes associated with WUE_p_ (Individual plant water use efficiency)

WUEp is calculated as the ratio between total plant dry mass (including roots) weight and total water use amount during the treatment period (planting to harvest) [34]. It can be observed from Table 4 that the higher WUE_p_ of most genotypes were observed under -WP or -W-P treatment. Most genotypes showed lower WUE_p_ under W-P treatment.

Under WP (control) treatment, chromosomes 5A, 7A, 6B, 4B, 3D, 6D, 7D, 2B, 3B, 4D, 3A, 2A, 2D, 5D, 5B and 4A had greater WUE_p_ and only chromosome 5A showed a significantly higher value than that of the two parents, while chromosome 1B had a little lower value than that of the two parents.

Under -WP conditions, only chromosomes 7D and 2D simultaneously possessed higher and lower WUE_p_ than that of the two parents, respectively.

Under W-P treatment, higher and lower WUE_p_ were observed on chromosomes 1A, 4B, 7A, 2B, 3D, 6B, 3B, 2D, 4D and 1D, 2A, 4A, 5A, 7B, 6A, 5D, 6D than that of the two parents, respectively. Only chromosome 1A had a significantly higher value than that of the two parents.

Under -W-P conditions, when compared with the two parents, higher WUE_p_ were observed on chromosomes 3D, 2A, 2B, 4B, 1D, 5A, 3B, 6A, 1A and 7A and lower values on chromosomes 5D and 5B; only the value of WUE_p_ on chromosomes 3D and 2A had any significant difference for the two parents.

### 2.5. Chromosomal location of the genes associated with PUT (phosphorus uptake) and PUE (phosphorus use efficiency)

PUT and PUE are two important indexes for measuring the phosphorus utilization capability. The definition and calculation have been described in the literature [17,18,35,36]. PUT and PUE of substitution lines under different conditions are shown in Table 5. All genotypes showed the highest PUT under WP (control) treatment and decreased under the other three stress conditions. However, the lowest value of PUE was observed under WP (control) treatment, except for 2D and the highest ones were found under W-P or -W-P treatments.

Under WP (control) treatment, chromosomes 3D, 5A, 3B, 5B and 6D showed higher and chromosomes 7B, 1A showed lower PUT than that of the two parents, and only the values of PUT on chromosome 3D, 5A and 3B showed any significant difference with the two parents. Chromosomes 7A, 2D, 7D, 4B, 5D and 7B might carry genes with positive effects on PUE, and chromosomes 3B, 2A, 1B, 3D, 3A, 6D, 5B, 4A, 4D, 6A, 1D, 5A, 2B and 6B might carry genes with negative effects on PUE compared with the two parents. However, only the values of PUE on chromosome 7A showed a significant difference compared with the two parents.

Under -WP treatment, chromosome 3B showed higher PUT than that of the two parents, but was significant different only for CS. Chromosomes 1A, 1D and 2A showed lower PUT than that of the two parents and only showed significant difference for ER. Chromosomes 1A, 2A, 1D, 2B, 7A, 7D, 5D, 6D, 6A, 3D, 4A, 3A and 6B were found with higher and chromosomes 2D,1B, 3B, 7B, and 4B with lower PUE than that of the two parents, respectively, but only the values of PUE on chromosomes 1A, 2A, 1D and 2D were significantly different for the two parents.

Under W-P conditions, chromosomes 5A, 1A, 6B, 4B, 2B, 4D, 5B and 3A might carry genes for PUT improvement; whereas 2A, 1B, 1D, 7D, 5D and 3D carry suppressor genes for PUT; but only chromosomes 5A and 1A had a significant difference compared with the two parents. Higher PUE were obtain from chromosomes 3D, 7A, 7D, 1B, 1D, 3B, 2A, 6D and lower ones were located on chromosomes 5A, 5B, 3A, 6B and 4A, respectively; only the value of PUE on chromosomes 3D, 7A, 7D, 1B, 1D, 5A and 5B showed a significant difference compared to the two parents.

Under -W-P treatment, all substitution lines showed higher PUT than that of the two parents except 5D, but only the value of PUT on chromosomes 4D, 3D showed a significant difference compared to the two parents; chromosome 5D had a lower PUT value than that of ER. The highest PUE was observed on ER, chromosomes 4D, 7B, 3D, 5D, 7D, 5A, 3A, 5B, 4A, 3B, 6B, 7A, 4B, 2B and 6A showed significant lower PUE than that of the two parents.

### 2.6. Relationship between WUE_l_ WUEp and PUE and related traits under different treatments

It can be seen from Table 6, that the phenotypic and genetic correlations between Pn and Tr were showed extremely significant positively correlation under WP (control), W-P, -WP treatment. Phenotypic correlations between Tr and WUE_l_ also showed significant or extremely significant negatively correlation under -W-P, W-P, -WP treatment. Genetic correlation between Tr and WUE_l_ showed extremely significant positive correlation under the four treatments.

Phenotypic and genetic correlation among DM, WUE_P_ and PUT all showed significant or extremely significant positively correlation under WP (control), W-P, and -WP treatments. There were significant or extremely significant and negative correlations between PUE and PUT under the four treatments.

### 3.1. Plant material and experimental design

Twenty-one substitution lines derived from the wheat varieties Chinese Spring and Egyptian Red, and their parents were used in this experiment. The parents were initially chosen for their differences in drought tolerance. The donor parent Egyptian Red, is a drought-tolerant wheat variety, whereas the recipient parent Chinese Spring, is a drought-sensitive variety. These substitution lines were often used to locate genes for major agriculture characteristics [23,25]. Chinese Spring was identified as a low phosphorus uptake variety [37], but the phosphorus uptake of Egyptian Red was not identified.

These materials were kindly provided by Dr Richard Richards (CSIRO Plant Industry, Australia). Uniform seeds were sterilized in 0.1% HgCl_2_ about 15 min, and rinsed for three times in distilled water. Seed germination was carried out in an incubator for two days and then they were transferred to plastic pots containing vermiculite. Seedlings were selected at the one-leaf stage and then they were cultured in triangular flasks. The triangular flasks were sealed with sealing film to prevent evaporation and wrapped with black plastic film to keep the roots in the dark. About a week after transplanting, the experiments were initiated on March 20, 2007, and for six weeks after the treatment application measurements were taken. The second repeated experiments were begun on March 27, 2007. All treatments were like in the first experiment. During the experiment period, the growth chamber had a day temperature range was 25–27 ºC and the night temperature range was 20–22 ºC, the relative humidity range was 60–70%. Artificial lighting (fluorescent tubes with 400 PFD) were used for seedling growth (14 h day/10 h night cycle, respectively).

The planting design was a randomized complete block with three replicates and six seedlings in each bottle. Two water regimes (W) and two phosphate treatment (P) was used. Four experimental treatments were conducted as: WP and W-P, which was control treatment (Hoagland solution) and P stress treatment (Hoagland solution with 1/2 P) respectively; While -WP, and -W-P which was osmotic treatment (Hoagland solution with 10% PEG), and osmotic and P stress treatment (Hoagland solution with 10% PEG and 1/2P), respectively. Culture solutions were changed once every 3–5 days during the growth period. The bottle positions were randomly switched every so often to decrease the differences in microclimates. Three seedling plants per bottle were randomly selected for measuring the investigated traits.

### 3.2. Determination of WUE and PUE

#### 3.2.1. Leaf water use efficiency (WUE_l_)

At wheat seedlings with six leaves, newly fully expanded leaves (the second leaf from the top) were selected for investigating leaf photosynthetic rate (Pn) and transpiration rate (Tr) with a portable photosynthesis system (LI-6400, Li-Cor, Lincoln, NE, USA). Nine leaves were measured for every treatment. Leaf water use efficiency (WUE_l_) was determined by Pn/Tr [38,39].

#### 3.2.2. Individual plant water use efficiency (WUE_p_)

After measuring WUE_l_, plant samples from three plants were collected from each bottle, then oven-dried at 80 ºC to constant weight and weighed with an analytical balance. The amount of water used during the plant growth period was determined by measuring water volume in bottle during the exchange of solution. WUE_p_ is calculated as the ratio between total plant dry mass (including roots) weight and total water use amount [34].

#### 3.2.3. PUT (phosphorus uptake) and PUE (phosphorus use efficiency)

The dried samples were milled and subsequently digested with concentrated H_2_SO_4_ and H_2_O_2_ for determining total P using the molybdate-blue colorimetric method. The calculation of PUT and PUE has been described in the literature [35,36].

### 3.3. Statistical analyses

Statistical analysis was performed using the SPSS 13.0 software. Duncan’s multiple range tests was employed for mean separation of each variable of investigated traits (photosynthetic rates, transpiration rates, leaf water use efficiency, dry mass, total water consumption, individual plant water use efficiency, phosphorous uptake, phosphorous use efficiency) among lines. Statistical difference in investigated traits under different treatments was assessed using one-way analysis of variance.

## 4. Conclusions

In this experiment, a set of Chinese Spring-Egyptian Red wheat substitution lines were used to locate the genes conferring WUE_l_, WUEp, PUE improvement or suppression on specific chromosomes at the seedling stage. Although the two parents were originally chosen because of their large differences in drought tolerance (Egyptian Red and Chinese Spring are drought tolerant and drought sensitive varieties, respectively), large differences between the two parents were also observed for the investigated traits. Egyptian Red showed greater WUE_l_ (except for W-P treatment) (Table 2), WUEp (Table 4), and PUT (Table 5) than Chinese Spring under all four treatments. However, Chinese Spring had higher PUE than that of Egyptian Red under WP, -WP and W-P treatment (Table 5).This might be a reflection of the fact that Egyptian Red is more tolerant to water and phosphorous deficiency than Chinese Spring.

It can be seen from Table 1 that Pn and Tr were more reduced by P stress than water stress. This is consistent with the results of [40–42], in which plant growth and Pn were limited by P deficiency and moderate water stress had no significant effects on these traits. It is also reported that plants may show reduced Tr and increased WUE_l_ to adapt to the stress condition [43]. Chromosomes 7D and 7A showed significantly increased Pn compared with CS under the four treatments. Chromosomes 6D, 4D, 6A and chromosome 2A showed higher Pn under WP, -WP, W-P treatment and WP, -WP, -W-P treatment, respectively; these chromosomes all showed significant increased Pn compared with CS. It may indicated that chromosomes 7D, 7A, 6D, 4D, 6A,2A may carry genes with positive effects on Pn. Chromosomes 5B and 3B carry genes with negative effects on Pn. A genome and chromosomes 3A, 3D, 4A and 4D were also reported to carry genes with increase Pn [24,25,44]. It seemed that genes with positive effects on Tr were carried by chromosomes 5D, 6D, 2D, 1D and 4A. The lower Tr values were mostly on the B genome.

From Table 2, most genotypes showed greater WUE_l_ under -WP conditions. The main reason due to the lower Tr rather than greater photosynthetic capacity, because Tr were greatly reduced under water stress condition. WUE_l_ on chromosomes 6B and 2 B were observed had higher value than two parents under WP (control), -WP and -W-P conditions. Chromosomes 1A, 7A, 7B and 3A also showed higher WUE_l_ under W-P, -W-P treatment, which indicated these chromosomes might carry genes associated with WUE_l_ improvement. While chromosomes 1D, 2D, 3D and 5D may carry genes with negative effects on WUE_l_ because they showed lower WUE_l_ than two parents under -WP, W-P, -W-P conditions. Chromosome 1A of wheat is also reported may carry genes involved in WUE_l._ [45], but observation differs from that in [25] that genes controlling water use efficiency were probably located on chromosomes 5A and 5D, which indicated that different location results may be obtained for different growth periods.

It could be seen from Table 3 that most genotypes had the highest DM under control treatment. TWC did no significant differences between control and W-P treatment, but was greatly reduced under PEG stress. Chromosomes 3D and 7D showed higher DM than the two parents under W-P, -W-P, WP and -WP, W-P treatments, respectively; chromosomes 1A, 7A, 2B, 3B and chromosomes 5A, 4B showed higher DM under W-P,-W-P and WP, -W-P treatments, respectively, which indicated that these chromosomes possibly carried genes for positive effects on DM, while chromosome 7B might carry genes with negative effects on DM, because it had lower values than the two parents under WP (control), W-P and -WP conditions. The results were consistent with suggestions that chromosomes 1A, 3D, and 5A carried genes with positive effects on DM [46]. Chromosomes 4D, 6D and 3D had higher TWC under -WP, W-P, -W-P treatments, this suggested that chromosome 4D, 6D and 3D possible carry genes increased TWC. Chromosomes 5D, 1B, 3A and chromosomes 4B, 2A, 4A and 5A may have deduced to be involved in reduced TWC because they showed lower values under WP (control), -WP, W-P and WP (control), -WP, and -W-P, respectively. This result was consistent with the finding of a previous study that chromosomes 2A, 3A, 4B and 5A may carry genes related to TWC [45].

As it shown from Table 4, most genotypes that showed the highest WUE_p_ under -WP treatment were partially due to the lower TWC. The other studies also showed that WUEp was greater in stressed treatments than in the well irrigated control [47,48]. Chromosomes 7A, 3D, 2B, 3B and 4B might carry genes for positive effects on WUE_p_, because they showed increased WUE_p_ under WP (control), W-P, and -W-P conditions (Table 4). This confirmed earlier observations that QTLs on chromosome 3B, 3D, 4A, 5B, 6D and 7A was vital for WUE_p_ when the recombinant inbred line (RIL) population (W7984 × Opata85) was used as material [44]. Chromosome 3D of wheat was also reported to carry genes with strong positive effects on vegetative WUEp [49]. Genes for decreased WUE_p_ might be carried by chromosome 5D because it showed lower WUE_p_ under W-P and -W-P treatments.

All substitute lines and their parents had the highest PUT and lowest PUE under WP (control) conditions (Table 5). For most substitute lines, water stress and nutrient stress all decreased PUT but to different degrees, and PUT was more reduced by P deficiency than water stress. On the contrary, P-deficiency induced a significant enhancement of PUE. Our findings are also consistent with [50–52], who reported that PUE were increased but there was decreased PUT under P deficiency. This result also confirmed former observation that decreasing water and nutrient supply decreased N, P and k uptake efficiency of different wheat species [31]. As Table 5 shows, genes with positive effects on PUT might be carried by chromosome 3B and chromosomes 5A, 5B because they showed increased PUT under WP (control), -WP, -W-P treatments and WP (control), W-P, -W-P treatments, respectively. The previous studies also revealed that the genes related to PUT had been located on chromosome 5A under P deficiency [16,18]. Chromosomes 7A, 7D carry genes for PUE improvement because they showed higher values than the two parents under WP, -WP and W-P conditions. Chromosomes 1B, 3B and chromosomes 4A, 3A, 5A, 5B, 6B showed lower PUE than that of the two parents under WP, -WP, -W-P and WP, W-P, -W-P treatments, respectively. Thus these chromosomes might carry genes with negative effects on PUE. This response was further confirmed by the results of [53,54], who found that chromosome 7A might carry gene(s) that could make wheat resistant to P deficiency, while chromosomes 1B and 3A might carry unfavorable gene(s) for this trait. It is also reported that chromosome 7A is closely related with PUE in wheat [23].

It can be seen from Table 6, WUE_l_ were mainly determined by Tr, because these two traits are closed correlated. The significant phenotypic and genetic positive correlations were found among DM, WUE_P_ and PUT. Therefore, DM may be as a good indicator for higher PUT and WUEp and also showed that water and phosphorus utilization was a related inheritance. The above results support the observations of location result from Tables 1–5, that these three traits have many similar location results under same treatment.

In conclusion, our results showed that chromosome 7A of substitution line were found to carry genes for increase in wheat WUE_l_, WUE_p_ and PUE. Other studies also showed that chromosome 7A may carry genes for adapting to stressful environments [29,55]. Therefore, chromosome 7A should be further studied and some of the important genes on this chromosome might be cloned and transferred by molecular genetic techniques. Gene locations on chromosomes of the investigated traits were not completely similar under different experimental conditions [56–59], this indicated that genetic mechanism(s) of WUE and PUE are highly complex. Thus, the chromosomal location of these traits in other growth periods and under different field conditions needs further study and we anticipate that very interesting data could be achieved through more verify, repeat experiments.

## Figures and Tables

**Table 1.** Photosynthetic rate (Pn) and transpiration rate (Tr) of the wheat substitution lines under different conditions (data are means ± SD of three replicates).

**Genotypes**	**Pn (μmol·m^2^·s^−1^)**			
	WP	-WP	W-P	-W-P

1A	5.2 ± 0.8AB	3.8 ± 0.8a	1.4 ± 0.1B	3.1 ± 0.03
2A	6.8 ± 0.4AB	5.2 ± 1.1Ab	1.4 ± 0.4B	3.9 ± 0.5
3A	7.9 ± 1.2AB	6.9 ± 1.2AB	1.7 ± 0.2B	2.7 ± 1.1
4A	9.5 ± 0.6AB	5.8 ± 0.2AB	2.1 ± 0.5B	3.5 ± 0.04
5A	7.2 ± 0.6AB	6.3 ± 0.4AB	1.9 ± 1.0B	2.2 ± 0.03b
6A	7.9 ± 0.03AB	8.1 ± 1.4AB	3.8 ± 0.9A	3.5 ± 0.2
7A	8.9 ± 0.3AB	8.8 ± 0.8AB	2.7 ± 0.4a	4.7 ± 0.8A
1B	4.4 ± 0.6Ab	3.9 ± 1.1	1.9 ± 0.7B	0.6 ± 0.AB
2B	2.5 ± 0.4	2.2 ± 0.4	1.8 ± 0.9B	1.9 ± 0.5B
3B	1.4 ± 0.1	2.5 ± 1.0	0.4 ± 0.1aB	1.5 ± 0.3aB
4B	2.0 ± 0.3	1.7 ± 0.01	1.7 ± 1.0B	1.6 ± 0.1aB
5B	1.8 ± 0.2	1.1 ± 0.2	1.7 ± 0.8B	1.1 ± 0.5AB
6B	4.5 ± 1.0Ab	4.2 ± 0.7	1.4 ± 0.3B	2.4 ± 0.02
7B	4.6 ± 1.0Ab	4.5 ± 0.2a	1.0 ± 0.1B	2.5 ± 0.9
1D	4.3 ± 0.5A	4 ± 0.3a	2.2 ± 0.4B	1.3 ± 0.5AB
2D	4.5 ± 0.7Ab	5.2 ± 0.6Ab	1.7 ± 0.7B	2.1 ± 0.2b
3D	5.9 ± 2.1AB	4.7 ± 0.9ab	1.8 ± 0.3B	0.7 ± 0.1AB
4D	4.4 ± 0.4Ab	4.7 ± 1.3ab	3.4 ± 0.6AB	0.9 ± 0.3AB
5D	4.6 ± 0.3Ab	4.4 ± 1a	1.2 ± 0.2B	1.8 ± 0.3aB
6D	5.8 ± 0.3AB	5.8 ± 1AB	2.9 ± 0.7AB	2.9 ± 1
7D	6.3 ± 0.9AB	5.8 ± 0.4AB	2.9 ± 0.4AB	4.4 ± 0.6a
CS	1.3 ± 0.5b	2.4 ± 0.1	1.5 ± 0.3B	2.9 ± 0.4
ER	2.8 ± 0.2a	2.6 ± 0.02	4.7 ± 0.7A	3.5 ± 0.5

**Genotypes**	**Tr (mmol·m^2^·s^−1^)**			
	WP	-WP	W-P	-W-P

1A	4.0 ± 0.7AB	3.4 ± 0.4AB	0.4 ± 0.04B	0.7 ± 0.04AB
2A	3.9 ± 0.9AB	1.9 ± 0.1ab	0.5 ± 0.07B	0.8 ± 0.2AB
3A	3.8 ± 0.2AB	3.0 ± 0.4AB	0.5 ± 0.1B	0.7 ± 0.1AB
4A	5.2 ± 1AB	2.0 ± 0.03aB	0.5 ± 0.02B	2.5 ± 0.01AB
5A	3.1 ± 0.7AB	2.7 ± 0.9AB	0.9 ± 0.2B	1.1 ± 0.1aB
6A	3.9 ± 0.7A	2.9 ± 0.8AB	1.2 ± 0.2aB	1.2 ± 0.01b
7A	5.1 ± 1.1AB	4.2 ± 0.06AB	0.7 ± 0.1B	1.1 ± 0.2aB
1B	1.5 ± 0.2	1.7 ± 0.4b	1.3 ± 0.01ab	0.4 ± 0.04AB
2B	1.1 ± 0.4	0.6 ± 0.2	0.8 ± 0.1B	0.8 ± 0.3AB
3B	0.5 ± 0.07	1.0 ± 0.3	0.5 ± 0.03B	0.8 ± 0.2AB
4B	1.2 ± 0.4	0.7 ± 0.02	1.0 ± 0.04B	0.6 ± 0.06AB
5B	0.8 ± 0.02	0.6 ± 0.2	0.6 ± 0.1B	0.6 ± 0.1AB
6B	1.5 ± 0.2	1.3 ± 0.7	0.4 ± 0.1B	0.5 ± 0.01AB
7B	1.7 ± 0.3	1.6 ± 0.2	0.3 ± 0.1B	0.8 ± 0.3AB
1D	2.6 ± 0.2Ab	1.7 ± 0.3	2.9 ± 0.8AB	2.8 ± 0.3AB
2D	2.0 ± 0.1a	2.3 ± 0.4AB	2.9 ± 0.8AB	4.2 ± 0.7AB
3D	2.3 ± 0.08a	1.7 ± 0.2	3.4 ± 0.06AB	1.2 ± 0.05B
4D	3.0 ± 0.9AB	1.3 ± 0.4	4.4 ± 0.5AB	1.3 ± 0.4b
5D	2.4 ± 0.2Ab	2.5 ± 0.8AB	2.1 ± 0.4A	2.4 ± 0.4Ab
6D	3.0 ± 0.3AB	2.4 ± 0.4AB	2.3 ± 0.7A	2.4 ± 0.2Ab
7D	2.9 ± 0.4AB	1.9 ± 0.3ab	1.9 ± 0.6A	1.2 ± 0.4b
CS	0.9 ± 0.2	0.9 ± 0.2	0.5 ± 0.03B	1.7 ± 0.6
ER	1.2 ± 0.004	0.8 ± 0.2	2.0 ± 0.5A	1.9 ± 0.3

**Note:** Letters A and a indicate a significantly different results than the parent China Spring at P = 0.01 and P = 0.05, respectively; Letters B and b indicate a significantly different result than the parent Egyptian Red at P = 0.01 and P = 0.05, respectively.

**Table 2.** Leaf water use efficiency (WUE_l_) of the wheat substitution lines under different conditions (data are means ± SD of three replicates).

**Genotypes**	**WUE_l_ (μmol·mmol^−1^)**			
	WP	-WP	W-P	-W-P
1A	1.3 ± 0.05B	1.1 ± 0.1AB	3.7 ± 0.4aB	4.8 ± 0.02AB
2A	1.8 ± 0.3	2.8 ± 0.8	2.9 ± 0.4	4.8 ± 0.8AB
3A	2.1 ± 0.4a	2.3 ± 0.07	3.2 ± 0.4	3.7 ± 0.5AB
4A	1.9 ± 0.5	2.9 ± 0.04	4.0 ± 0.2AB	1.4 ± 0.01
5A	2.3 ± 0.3a	2.4 ± 0.7	2.0 ± 0.3	2.0 ± 0.2
6A	2.1 ± 0.4	2.8 ± 0.3	3.4 ± 1b	2.8 ± 0.2AB
7A	1.8 ± 0.3	2.3 ± 0.2	3.7 ± 0.2aB	4.1 ± 0.4AB
1B	2.9 ± 0.04A	2.4 ± 0.03	1.5 ± 0.5Ab	1.7 ± 0.6
2B	2.5 ± 0.6A	3.4 ± 0.3	2.3 ± 0.3	2.4 ± 0.a
3B	2.9 ± 0.1A	2.5 ± 0.3	0.7 ± 0.2AB	2.0 ± 0.4
4B	1.7 ± 0.4	2.4 ± 0.06	1.7 ± 1A	2.5 ± 0.4a
5B	2.2 ± 0.2a	2.1 ± 0.3b	2.7 ± 1	1.7 ± 0.6
6B	2.9 ± 0.3A	3.5 ± 1.2	3.2 ± 0.4	4.3 ± 0.02AB
7B	2.7 ± 0.1A	2.9 ± 0.3	3.3 ± 0.3	3.1 ± 0.3AB
1D	1.6 ± 0.1b	2.4 ± 0.5	0.8 ± 0.1AB	0.5 ± 0.1AB
2D	2.3 ± 0.3a	2.3 ± 0.1b	0.6 ± 0.3AB	0.5 ± 0.05AB
3D	2.6 ± 1A	2.8 ± 0.2	0.5 ± 0.1AB	0.6 ± 0.01AB
4D	1.5 ± 0.3b	3.6 ± 0.01	0.8 ± 0.04AB	0.7 ± 0.2AB
5D	1.9 ± 0.2	1.7 ± 0.6aB	0.6 ± 0.1AB	0.7 ± 0.1AB
6D	1.9 ± 0.3	2.4 ± 0.06	1.3 ± 0.4AB	1.2 ± 0.5
7D	2.2 ± 0.02a	3.1 ± 0.3	1.6 ± 0.8Ab	3.5 ± 0.6AB
CS	1.4 ± 0.2B	2.9 ± 0.6	2.9 ± 0.6	1.7 ± 0.3
ER	2.4 ± 0.2A	3.3 ± 0.9	2.4 ± 0.3	1.9 ± 0.6

**Note:** A and a indicate a significantly different performance than the parent China Spring at P = 0.01 and P = 0.05, respectively; B and b indicate a significantly different performance than the parent Egyptian Red at P = 0.01 and P = 0.05, respectively.

**Table 3.** DM (dry mass), TWC (total water consumption) of the wheat substitution lines under different conditions (data are means ± SD of three replicates).

**Genotypes**	**DM (g/plant)**			
	WP	-WP	W-P	-W-P

1A	0.13 ± 0.03B	0.1 ± 0.008B	0.26 ± 0.1AB	0.14 ± 0.01
2A	0.16 ± 0.003b	0.15 ± 0.01b	0.11 ± 0.01	0.17 ± 0.007a
3A	0.19 ± 0.006	0.15 ± 0.02b	0.15 ± 0.01	0.11 ± 0.08
4A	0.19 ± 0.001	0.15 ± 0.01b	0.11 ± 0.007	0.11 ± 0.01
5A	0.29 ± 0.03A	0.17 ± 0.007	0.16 ± 0.01	0.15 ± 0.05
6A	0.14 ± 0.01B	0.13 ± 0.03b	0.14 ± 0.02	0.15 ± 0.03
7A	0.24 ± 0.07a	0.18 ± 0.01	0.22 ± 0.04Ab	0.16 ± 0.01a
1B	0.11 ± 0.03B	0.16 ± 0.004	0.14 ± 0.02	0.13 ± 0.01
2B	0.22 ± 0.04	0.17 ± 0.02	0.21 ± 0.05a	0.19 ± 0.03A
3B	0.21 ± 0.03	0.19 ± 0.02a	0.18 ± 0.02	0.17 ± 0.06a
4B	0.25 ± 0.02A	0.16 ± 0.11	0.19 ± 0.02	0.14 ± 0.04
5B	0.24 ± 0.05a	0.14 ± 0.02b	0.15 ± 0.004	0.07 ± 0.006
6B	0.22 ± 0.01	0.13 ± 0.03b	0.17 ± 0.03	0.1 ± 0.02
7B	0.12 ± 0.03B	0.10 ± 0.05B	0.13 ± 0.006	0.1 ± 0.04
1D	0.13 ± 0.03B	0.14 ± 0.04b	0.15 ± 0.007	0.17 ± 0.03a
2D	0.19 ± 0.03	0.09 ± 0.02B	0.16 ± 0.03	0.15 ± 0.008a
3D	0.25 ± 0.07A	0.22 ± 0.03A	0.2 ± 0.03	0.2 ± 0.007A
4D	0.21 ± 0.01	0.16 ± 0.03	0.19 ± 0.02	0.12 ± 0.03
5D	0.19 ± 0.03	0.18 ± 0.02	0.12 ± 0.03	0.05 ± 0.01b
6D	0.22 ± 0.02	0.15 ± 0.05b	0.16 ± 0.01	0.11 ± 0.07
7D	0.23 ± 0.02a	0.24 ± 0.07A	0.18 ± 0.03	0.1 ± 0.05
CS	0.15 ± 0.06b	0.12 ± 0.02B	0.15 ± 0.03	0.08 ± 0.03
ER	0.24 ± 0.04a	0.23 ± 0.01A	0.16 ± 0.01	0.13 ± 0.04

**Genotypes**	**TWC (g/plant)**			
	WP	-WP	W-P	-W-P

1A	91.7 ± 10.2AB	58.7 ± 5.3b	103.6 ± 6.3	60.9 ± 1.3
2A	85.2 ± 0.04AB	61.9 ± 6.4	98.8 ± 7.8	59.0 ± 5.4
3A	95.8 ± 0.03AB	55.2 ± 7.7aB	95.6 ± 8.8	77.8 ± 2.9AB
4A	108.5 ± 5.5AB	59.8 ± 1.9b	103.4 ± 0.2	59.8 ± 3.8
5A	93.8 ± 4.4AB	57.4 ± 1.9ab	137 ± 0.1AB	60.5 ± 4.6
6A	97.0 ± 0.8AB	66.6 ± 1.3	104.9 ± 5.4	65.7 ± 5.2
7A	96.3 ± 0.7AB	62.0 ± 3.5	118.0 ± 6.1AB	69.7 ± 1.4
1B	108.8 ± 8.3AB	58.4 ± 1.5ab	95.4 ± 0.1	72.6 ± 1.8b
2B	100.6 ± 0.6AB	62.2 ± 4.3	110.1 ± 0.5b	67.4 ± 6.1
3B	100.9 ± 6.6AB	63.7 ± 0.3	99.5 ± 0.06	71.3 ± 6.7b
4B	105.4 ± 3.8AB	64.4 ± 6.4	98.6 ± 0.1	53.4 ± 5.5
5B	128.1 ± 9.8	69.4 ± 1.2	90.7 ± 0.08a	67.2 ± 2.7
6B	93.6 ± 9.3AB	61.4 ± 6.8	98.5 ± 7.6	73.2 ± 8.2aB
7B	86.5 ± 0.1AB	51.8 ± 8.1AB	100.1 ± 0.5	65.6 ± 6.8
1D	101.2 ± 5.8AB	58.1 ± 9.1ab	128.9 ± 7.8AB	70.2 ± 3.8
2D	103.4 ± 5.3AB	61.2 ± 0.7	98.1 ± 8.2	72.4 ± 3.8b
3D	109.2 ± 4.4AB	74.5 ± 0.3	108.3 ± 2.8b	65.4 ± 5.9
4D	102.4 ± 4.5AB	73.3 ± 6.9	118.6 ± 0.2AB	75.8 ± 6.6aB
5D	105.9 ± 11.1AB	58.9 ± 0.4b	90.9 ± 4.8a	68.4 ± 4.6
6D	97.5 ± 4.5AB	80.9 ± 6a	124.1 ± 4.0AB	71.9 ± 1.1b
7D	102.5 ± 3.2AB	65.9 ± 9.5	110.1 ± 8.9b	64.6 ± 8.7
CS	128.7 ± 8.2	68.8 ± 4.2	103.4 ± 5.0	62.5 ± 0.8
ER	139.5 ± 0.1a	70.8 ± 0.2	96.9 ± 0.78	60.6 ± 7.2

**Note:** A and a indicate a significantly different performance than the parent China Spring at P = 0.01 and P = 0.05, respectively; B and b indicate a significantly different performance than the parent Egyptian Red at P = 0.01 and P = 0.05, respectively.

**Table 4.** Individual plant water use efficiency (WUE_p_) of the wheat substitution lines under different conditions. (Data are means ± SD of three replications).

**Genotypes**	**Treatment: WUEp (mg/g)**			
	WP	-WP	W-P	-W-P
1A	1.4 ± 0.05	1.7 ± 0.3B	2.5 ± 0.3AB	2.3 ± 0.3A
2A	1.9 ± 0.03	2.5 ± 0.4ab	1.1 ± 0.03	2.9 ± 0.3bA
3A	1.9 ± 0.06	2.8 ± 0.03A	1.6 ± 0.01	1.4 ± 0.1b
4A	1.7 ± 0.3	2.5 ± 0.3ab	1.1 ± 0.07	1.9 ± 0.1
5A	3.1 ± 0.2AB	3 ± 0.02A	1.2 ± 0.07	2.4 ± 0.4A
6A	1.4 ± 0.6	1.9 ± 0.5B	1.3 ± 0.01	2.3 ± 0.02A
7A	2.5 ± 0.7A	2.9 ± 0.4A	1.9 ± 0.8	2.2 ± 0.2A
1B	1 ± 0.7	2.7 ± 0.1A	1.5 ± 0.2	1.8 ± 0.2
2B	2.2 ± 0.4a	2.8 ± 0.5A	1.9 ± 0.5	2.8 ± 0.6A
3B	2.1 ± 0.4a	3 ± 0.4A	1.8 ± 0.2	2.4 ± 0.2A
4B	2.4 ± 0.3a	2.5 ± 0.3ab	1.9 ± 0.2a	2.7 ± 0.4A
5B	1.8 ± 0.6	1.9 ± 0.3B	1.6 ± 0.04	1.0 ± 0.05B
6B	2.4 ± 0.8a	2.1 ± 0.04B	1.8 ± 0.01	1.3 ± 0.5b
7B	1.4 ± 0.3	1.9 ± 0.3B	1.3 ± 0.3	1.6 ± 0.07
1D	1.3 ± 0.3	2.4 ± 0.3aB	1.1 ± 0.01	2.4 ± 0.1A
2D	1.8 ± 0.2	1.5 ± 0.3B	1.7 ± 0.1	2.1 ± 0.4A
3D	2.3 ± 0.6a	2.9 ± 0.4A	1.8 ± 0.3	2.9 ± 0.4bA
4D	2 ± 0.2	2.1 ± 0.01B	1.6 ± 0.07	1.6 ± 0.05
5D	1.8 ± 0.7	3.1 ± 0.4A	1.3 ± 0.4	0.7 ± 0.1B
6D	2.3 ± 0.09a	1.9 ± 0.3B	1.3 ± 0.2	1.5 ± 0.7b
7D	2.3 ± 0.3a	3.6 ± 0.4A	1.6 ± 0.01	1.6 ± 0.08
CS	1.2 ± 0.04	1.7 ± 0.4B	1.4 ± 0.01	1.2 ± 0.3B
ER	1.7 ± 0.3	3.3 ± 0.2A	1.6 ± 0.1	2.2 ± 0.2A

**Note:** A and a indicate significantly different performance than the parent China Spring at P = 0.01 and P = 0.05, respectively; B and b indicate a significantly different performance than the parent Egyptian Red at P = 0.01 and P = 0.05, respectively.

**Table 5.** PUT (phosphorous uptake) and PUE (phosphorous use efficiency) of the wheat substitution lines under different conditions (data are means ± SD of three replicates).

**Genotypes**	**PUT (mg/plant)**			

	WP	-WP	W-P	-W-P

1A	3.72 ± 0.2B	1.35 ± 0.02B	3.28 ± 0.9Ab	1.11 ± 0.07
2A	6.89 ± 0.4A	2.21 ± 0.2B	1.32 ± 0.1b	1.5 ± 0.05
3A	7.03 ± 0.02A	3.08 ± 0.05B	2.49 ± 0.5	1.51 ± 0.7
4A	6.88 ± 0.15A	3.00 ± 0.09B	1.89 ± 0.3	1.43 ± 0.05
5A	9.69 ± 0.17AB	3.71 ± 0.7B	3.31 ± 0.4Ab	2.07 ± 0.6ab
6A	4.71 ± 1.47B	2.48 ± 0.3B	1.88 ± 0.2	1.57 ± 0.4
7A	5.48 ± 1.32b	3.17 ± 0.2B	1.94 ± 0.3	1.75 ± 0.1
1B	4.79 ± 0.83B	4.84 ± 0.7A	1.35 ± 0.09b	1.29 ± 0.1
2B	7.18 ± 0.27A	2.84 ± 0.2B	2.72 ± 0.7a	2.00 ± 0.3a
3B	9.42 ± 0.83Ab	5.71 ± 0.7A	2.04 ± 0.1	1.99 ± 0.9a
4B	6.34 ± 0.62a	4.00 ± 0.1ab	2.73 ± 0.4a	1.6 ± 0.3
5B	8.67 ± 0.03A	2.97 ± 0.6B	2.55 ± 0.3	0.93 ± 0.1
6B	7.17 ± 0.01A	2.62 ± 0.5B	2.79 ± 0.7a	1.12 ± 0.4
7B	3.29 ± 0.5B	2.69 ± 0.9B	1.84 ± 0.2	1.87 ± 0.9a
1D	4.51 ± 0.5B	2.06 ± 0.5B	1.61 ± 0.02	1.48 ± 0.3
2D	4.65 ± 1.4B	3.12 ± 1.6B	2.28 ± 0.5	1.52 ± 0.004
3D	10.09 ± 1.1AB	4.29 ± 0.1A	1.71 ± 0.3	3.24 ± 0.02AB
4D	7.08 ± 0.3a	3.51 ± 0.5B	2.69 ± 0.4a	4.18 ± 0.4AB
5D	5.21 ± 0.6a	3.27 ± 0.2B	1.67 ± 0.3	0.75 ± 0.2
6D	8.21 ± 0.2A	2.80 ± 0.7B	1.93 ± 0.2	0.85 ± 0.5
7D	5.84 ± 1.2a	4.20 ± 1.6A	1.62 ± 0.3	1.63 ± 0.8
CS	4.39 ± 0.8B	2.41 ± 0.2B	1.79 ± 0.4	0.69 ± 0.3
ER	7.30 ± 0.08A	5.54 ± 0.4A	2.28 ± 0.3	0.86 ± 0.3

**Genotypes**	**PUE (g/g)**			

	WP	-WP	W-P	-W-P

1A	34.1 ± 3.5	74.5 ± 2.4AB	78.6 ± 0.5b	123.5 ± 1.3AB
2A	23.6 ± 0.9ab	68.9 ± 1.0AB	85.3 ± 1.9B	112.5 ± 3.2B
3A	26.5 ± 0.4	49.4 ± 2.7	61.5 ± 6.5A	72.9 ± 0.4AB
4A	27.5 ± 0.4	49.5 ± 2.7	62.9 ± 6.0A	77.4 ± 7.2AB
5A	30.4 ± 1.2	46.8 ± 3.4	48.0 ± 2.8AB	69.9 ± 4AB
6A	29.5 ± 3.3	52.3 ± 2.9	72.1 ± 2.8	94.4 ± 6.1AB
7A	43.1 ± 1.4ab	58.1 ± 0.4b	116.0 ± 4.2AB	88.6 ± 0.9AB
1B	23.8 ± 0.9ab	32.7 ± 2.9a	104.0 ± 4.2AB	103.1 ± 2.6B
2B	30.5 ± 2.5	60.6 ± 5.6B	76.1 ± 1.4	93.9 ± 5.1AB
3B	22.3 ± 0.5ab	33.9 ± 0.1a	86.3 ± 2.2B	84.1 ± 7.3AB
4B	39.5 ± 0.4	39.8 ± 4.6	69.8 ± 3.6A	89.8 ± 6.1AB
5B	27.3 ± 2.9	46.1 ± 1.3	57.1 ± 5.3AB	73.9 ± 2.8AB
6B	30.9 ± 0.7	49.0 ± 1.5	62.6 ± 6.1A	85.1 ± 6.4AB
7B	36.5 ± 1.6	38.3 ± 2.1	69.8 ± 3.6A	54.4 ± 5.3AB
1D	29.7 ± 1.4	66.4 ± 2.1AB	89.9 ± 3.5aB	114.7 ± 2.3B
2D	41.1 ± 3.1	28.5 ± 4.2Ab	72.3 ± 2.7a	101.4 ± 5B
3D	25.1 ± 2.3a	51.3 ± 3.2	116.0 ± 1.4AB	60.2 ± 1.6AB
4D	28.9 ± 0.3	44.3 ± 1.3	72.5 ± 2.7a	28.7 ± 3.9AB
5D	37.5 ± 3.9	56.3 ± 5.1b	71.0 ± 3.2	63.6 ± 3.8AB
6D	26.9 ± 0.1	53.6 ± 1.8	85.2 ± 1.8B	126.4 ± 8.7AB
7D	39.5 ± 3.1	56.9 ± 1.8b	107.9 ± 5.6AB	64.2 ± 3.3AB
CS	34.2 ± 3.2	48.1 ± 5.3	80.9 ± 0.3B	109.4 ± 5.1B
ER	33.3 ± 2.7	41.6 ± 2.7	68.6 ± 4.0A	152.7 ± 4.8A

**Note:** A and a indicate a significant difference with the parent China Spring at P = 0.01 and P = 0.05, respectively; B and b indicate a significant difference with the parent Egyptian Red at P = 0.01 and P = 0.05, respectively.

**Table 6.** Significance compare between phenotypic correlation and genetic correlation between WUE_l_, WUEp, PUE and related traits.

**Treatment**	**Phenotypic Correlation Genetic Correlation**															
WP		Pn	Tr	WUE_l_	DM	TWC	WUE_p_	PUT		Pn	Tr	WUE_l_	DM	TWC	WUE.p	PUT
	Tr	**							Tr	**						
	WUE_l_								WUE_L_		-**					
	DM								DM							
	TWC	-*	-*						TWC	-*	-*					
	WUE_p_				**				WUE_P_				**			
	PUT				**		**		PUT				**		**	
	PUE							-*	PUE			-*				-**

-WP		Pn	Tr	WUE_l_	DM	TWC	WUE_p_	PUT		Pn	Tr	WUE_l_	DM	TWC	WUE.p	PUT
	Tr	**							Tr	**						
	WUE_l_		-*						WUE_l_		-**					
	DM								DM			*				
	TWC								TWC			**				
	WUE_p_				**				WUEP				**			
	PUT				**		**		PUT		-*		**		**	
	PUE							-**	PUE		*					-**

W-P		Pn	Tr	WUEl	DM	TWC	WUE_p_	PUT		Pn	Tr	WUEl	DM	TWC	WUE.p	PUT
	Tr	*							Tr	**						
	WUE_l_		-**						WUE_l_		-**					
	DM								DM							
	TWC								TWC							
	WUE_p_				**				WUEP				**			
	PUT				**		*		PUT				*		**	
	PUE							-**	PUE							-**

-W-P		Pn	Tr	WUE_l_	DM	TWC	WUE_p_	PUT		Pn	Tr	WUE_l_	DM	TWC	WUE.p	PUT
	Tr								Tr							
	WUE_l_	**	-**						WUE_l_	**	-**					
	DM								DM							
	TWC								TWC	-*						
	WUE_p_				**				WUEP				**	-*		
	PUT				*				PUT	-*						
	PUE							-**	PUE							-**

**Note:** ** and * indicated positive correlation is significant at 0.01, 0.05 probability level, respectively;

–** and –* indicated negative correlation is significant at 0.01, 0.05 probability level, respectively.

## Abbreviations:

CS: Chinese Spring
ER: Egyptian Red
WUE: water use efficiency
PUE: phosphorus use efficiency
WUE_l_: leaf water use efficiency
WUE_p_: individual plant water use efficiency
Pn: net photosynthetic rate
Tr: transpiration rate
DM: dry mass
PUT: phosphorus uptake
TWC: total water consumption
PEG: polyethylene glycol
WP: Hoagland solution
W-P: Hoagland solution with 1/2P
-WP: Hoagland solution with 10% PEG
-W-P: Hoagland solution with 1/2P and 10% PEG
